# Corridor and real-time 6-minute walk tests in healthy young adults: A randomized cross-over study^☆^

**DOI:** 10.1016/j.jtumed.2024.05.005

**Published:** 2024-05-15

**Authors:** Mohamed A. Al Maghraby, Ali M. Alshami, Qassim I. Muaidi, Turki S. Abualait, Matar A. Alzahrani, Sultan S. Alotaibi, Hassan M. Alamir, Mashari A. AlOudah, Abdulmajeed A. Aljiry, Hassan S. Alhijji, Abdullah S. Alomar, Mohammed Al Ensaif

**Affiliations:** a Department of Physical Therapy, Imam Abdulrahman Bin Faisal University, Dammam, KSA; b Department of Physical Therapy, Prince Sultan Cardiac Center, Al Ahsa, KSA; c Department of Physical Therapy, Imam Abdulrahman Al Faisal Hospital, Dammam, KSA; d Department of Physical Therapy, Al Fateh Sports Club, Al Ahsa, KSA; e Physical Therapy and Rehabilitation Department, Al Jafer General Hospital, Al Ahsa, KSA; f Rehabilitation Department, Mouwsat Medical Group, Dammam, KSA; g Department of Physical Therapy, Mouwasat Medical Group, Qatif, KSA

**Keywords:** اختبار المشي لمدة ست دقائق, المسافة, الوظيفة, السرعة, جهاز المشي, الواقع الافتراضي, 6-minute walk test (6MWT), Distance, Function, Speed, Treadmill, Virtual reality

## Abstract

**Objective:**

Cardiopulmonary endurance is important for comfortably participating in activities of daily living. Exercise tests, such as the 6-minute walk test (6MWT), are commonly used to evaluate cardiopulmonary endurance. We investigated the effects of the Gait Real-Time Analysis Interactive Lab (GRAIL)- and corridor-based 6MWTs on functional performance.

**Methods:**

Thirty healthy men were randomly divided into two groups. Group A participants performed a corridor-based 6MWT, followed by a washout period (1 h). Subsequently, they performed the GRAIL-based 6MWT. Group B participants performed the tests in the reverse order of that performed by Group A participants.

**Results:**

The corridor-based 6MWT resulted in significantly higher 6MW distance and 6MW speed than the GRAIL-based 6MWT. No significant differences were observed between the two groups in any of the following secondary outcomes: systolic blood pressure, diastolic blood pressure, oxygen saturation, heart rate, dyspnea, and overall fatigue. A strong positive correlation was observed between the 6MW distance and 6MW speed.

**Conclusion:**

The corridor- and GRAIL-based 6MWT should not be used interchangeably.

## الملخص

**أهداف البحث:**

التحمل القلبي الرئوي مهم للمشاركة بشكل مريح في أنشطة الحياة اليومية. تستخدم اختبارات التمرين، مثل اختبار المشي لمدة ست دقائق، بشكل شائع لتقييم القدرة على التحمل القلبي الرئوي. نحن نهدف إلى دراسة تأثيرات اختبار المشي لمدة ست دقائق القائم على الممر والمعتمد على مختبر تحليل المشية في الوقت الحقيقي التفاعلي على الأداء الوظيفي.

**طريقة البحث:**

تم تقسيم ثلاثين رجلا أصحاء بشكل عشوائي إلى مجموعتين. أجرى المشاركون في المجموعة (أ) اختبار المشي لمدة ست دقائق على الممر، تليها فترة الراحة (ساعة واحدة)، وبعد ذلك، قاموا بإجراء اختبار المشي في الوقت الحقيقي للتحليل التفاعلي القائم على المشي لمدة ست دقائق. في المجموعة (ب)، أجرى المشاركون الاختبارات بتسلسل عكسي لتلك التي أجراها المشاركون في المجموعة (أ).

**النتائج:**

أدى اختبار المشي لمدة ست دقائق القائم على الممر إلى زيادة كبيرة في مسافة ست دقائق سيرا على الأقدام (6 ميجاوات) وسرعة المشي لمدة ست دقائق مقارنة باختبار المشي لمدة ست دقائق القائم على مختبر التحليل التفاعلي في الوقت الحقيقي. لم يلاحظ أي فروق ذات دلالة إحصائية بين المجموعتين في أي من النتائج الثانوية التالية: ضغط الدم الانقباضي، وضغط الدم الانبساطي، وتشبع الأكسجين، ومعدل ضربات القلب، وضيق التنفس، والتعب العام. ولوحظ وجود علاقة إيجابية قوية بين مسافة اختبار المشي البالغة ست دقائق وسرعة اختبار المشي لمدة ست دقائق.

**الاستنتاجات:**

لا ينبغي استخدام اختبار المشي لمدة ست دقائق القائم على الممر واختبار المشي في الوقت الحقيقي للتحليل التفاعلي القائم على المختبر لمدة ست دقائق بشكل تبادلي.

## Introduction

Cardiopulmonary endurance is important for comfortably participating in activities of daily living.^1^ Exercise tests, such as the 6-minute walk test (6MWT), are commonly used to evaluate cardiopulmonary endurance.^1^ The 6MWT is a submaximal self-paced test of exercise and functional capacity and measures the maximum distance that a person can normally walk in 6 min.^1^^,^^2^ The 6MWT is a validated measure of functional exercise capacity in various populations because it is simple, inexpensive, and requires minimal equipment and space. The 6MWT results reflect the functioning of multiple body systems (cardiovascular, respiratory, and musculoskeletal) and provide valuable insights into a patient's overall functional capacity.^1^^,^^2^

According to the guidelines of the American Thoracic Society (ATS), the 6MWT should be performed in an enclosed corridor with at least a 30 m (100 feet) hallway to minimize turns and maximize walking distance.^2^ However, such hallways are not always feasible in clinical or research settings, and measurement of physiological responses during the test is difficult. Therefore, alternative methods have been used in which less space is used to monitor vital signs^1^; for example, studies have been conducted to compare the results of the treadmill 6MWT with those of the corridor 6MWT. However, these studies are few and have yielded mixed results.^1^

Self-paced treadmill walking offers a natural way of controlling and varying walking speed that results in a more natural gait pattern than that for fixed-speed treadmill walking.^3^ Moreover, virtual reality during treadmill walking is becoming increasingly common in rehabilitation because it provides an engaging and real-life environment.^4^^,^^5^ The Gait Real-Time Analysis Interactive Lab (GRAIL) system provides self-paced treadmill walking with features such as virtual reality and three-dimensional (3D) motion capture to analyze gait patterns during gait. The GRAIL system overcomes the limitations of corridor and fixed-speed treadmill walking.^5^ The GRAIL-based 6MWT results were found to be comparable to those of the corridor-based 6MWT in patients and healthy individuals.^5^

Evaluating the reproducibility of the GRAIL-based 6MWT results and comparing them with the corridor-based 6MWT results are essential; however, the use of GRAIL for 6MWT in adults remains understudied. The only study that examined GRAIL-based 6MWT results versus corridor-based 6MWT results was conducted in patients with chronic obstructive pulmonary disease (COPD) and healthy elderly individuals.^5^

Therefore, in this study, we compared the effects of GRAIL- and corridor-based 6MWT on walking distance and physiological responses in young healthy individuals. We hypothesized that the GRAIL-based 6MWT would result in favorable outcomes in terms of walking distance and physiological responses compared with the corridor-based 6MWT. Initially, we hypothesized that the motivational benefits of GRAIL observed in exercise tasks (based on theory) could indirectly translate into improved performance on the 6MWT. We also considered studies on self-paced treadmill walking,^6^ assuming that they would offer supporting evidence. However, the connection to gait analysis in the context of the 6MWT may not be directly applicable.

## Materials and Methods

### Study design

This was a single-center, randomized, two-period, two-sequence cross-over study. The tests were conducted between 8:30 and 11:30 a.m. to minimize the effects of diurnal biological variations.^7^

### Sample size calculation

The sample size was determined using G∗Power version 3.1 (Düsseldorf, Germany), and the difference between two means of the 6MWT distances previously reported (corridor-based 6MWT = 668.8 ± 73.6 m; GRAIL-based 6MWT = 692.3 ± 62.0 m) was used for the calculation.^5^ This resulted in an effect size of 0.53. A paired *t*-test was used with an alpha level of 0.05 and a power of 0.80. A minimum sample size of 24 was required. Assuming a 20% dropout rate, 30 participants were included in this study.

### Participants

Healthy male adults who were nonsmokers, non-obese (body mass index [BMI] <30 kg/m^2^), or able to ambulate independently were included in the study. Participants were excluded if their resting heart rate (HR), systolic blood pressure (SBP), and diastolic blood pressure (DBP) were >120 bpm, >180 mm Hg, and >100 mm Hg, respectively, on the day of testing or if they had cognitive disorders, were unable to walk independently, or were using medications that influenced vital signs.^8^ A convenience sample was obtained from students enrolled in the physical therapy program. All participants signed a consent form and were informed of the purpose and detailed procedures of the study if they met the inclusion criteria and agreed to participate.

### Randomization

**Figure 1:**
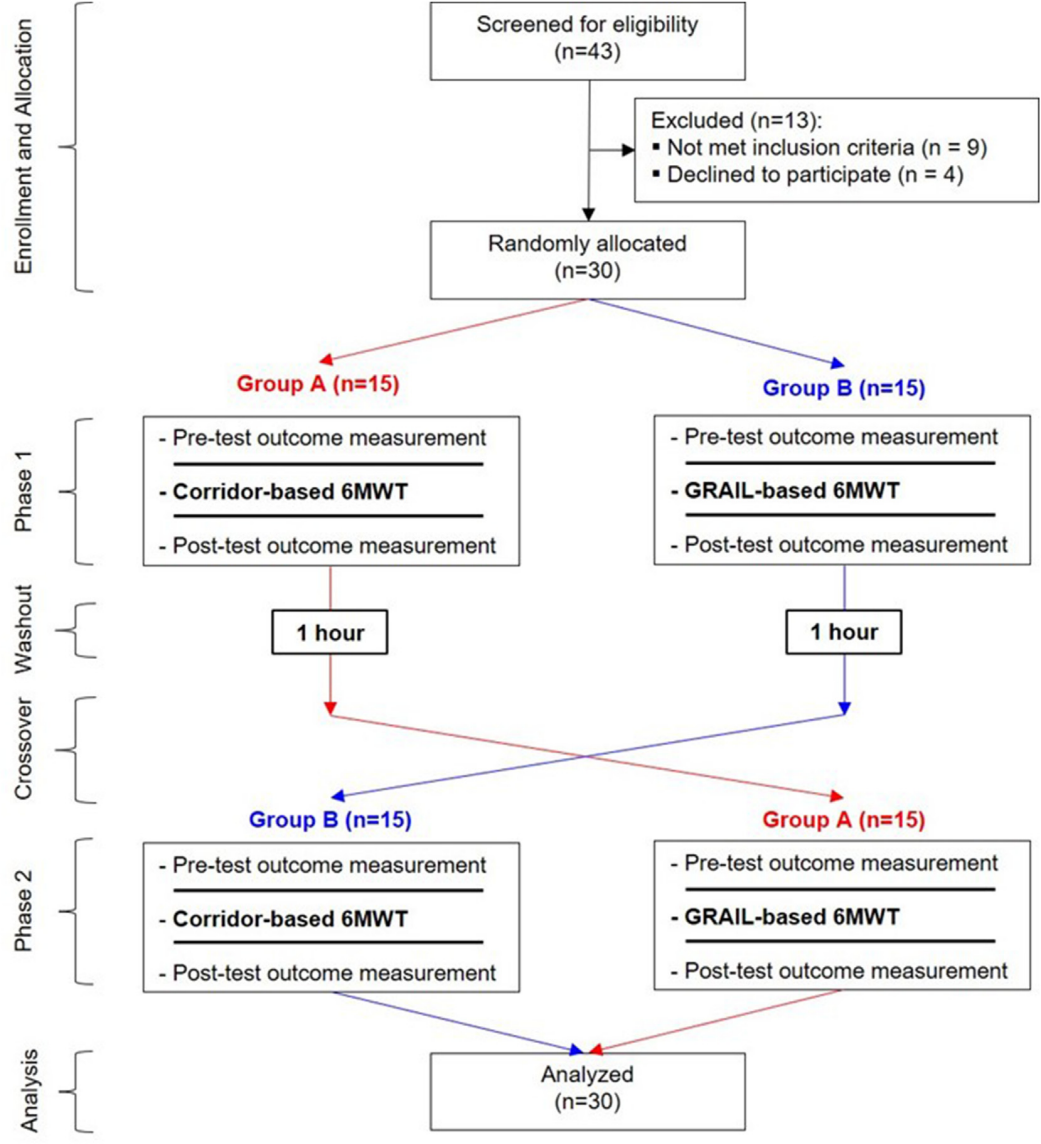
Flow chart of the study design. 6MWT: 6-minute walk test.

Before initiating the study, an allocation sequence of 30 numbers was generated, which were uniquely randomized into two groups of equal numbers (15 in each group) in a parallel design (1:1 ratio): Group A and Group B. In this cross-over study, the participants in Group A performed the corridor-based 6MWT, followed by a washout period of 1 h, and subsequently performed the GRAIL-based 6MWT. Group B participants performed the GRAIL-based 6MWT; after the 1 h washout period, they performed the corridor-based 6MWT (Figure 1).

### Testing

All of the participants were instructed to perform the 6MWT before the actual measurement to familiarize themselves with the testing procedure. Practice training was performed for the GRAIL-based 6MWT but not for the corridor-based 6MWT. After the practice test, the participants were asked to wait for at least 1 h before the actual test started.^2^ Each participant performed both the corridor- and GRAIL-based 6MWT three times each (with 1-h rest between each test). The best distance covered was recorded for analysis. The tests were performed on a single day, and each session lasted approximately 3 h. All tests were performed by the same researcher.

*Corridor-based 6MWT*. This test was performed in an undisturbed 70-m corridor according to ATS guidelines.^2^ The 6MWT results were adjusted using a digital stopwatch. The participants were instructed to walk as far as possible within 6 min. They were allowed to stop, rest, and continue, as they wanted. The countdown time (within the allowed 6 min) was maintained even during the resting period. A chair was placed in the corridor for rest. Markers were located along the walking course at 5-m intervals. At the end of the testing period (6 min), the total distance walked by the participants was measured to the nearest meter. The walking speed was measured using the following equation: distance (m)/time (s).

**Figure 2:**
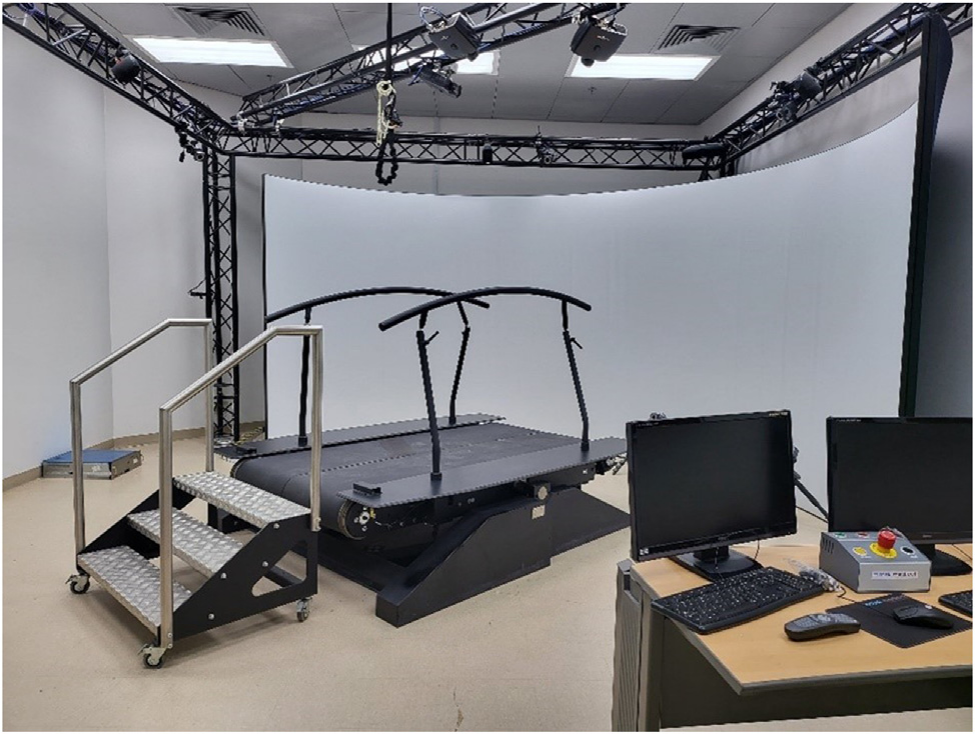
Gait Real-Time Analysis Interactive Lab system.

*GRAIL-based 6MWT*. A GRAIL (Motekforce Link, Amsterdam, the Netherlands) with virtual reality was used as a self-paced treadmill. The GRAIL demonstrated good validity and reliability (intraclass correlation coefficient: 0.65–0.8) in assessing the 6MW distance (6MW_D_).^5^ The GRAIL is a synchronized system that consists of an instrumented dual-belt treadmill with a self-paced option that is integrated with a 3D motion-capture system along with three video cameras (Figure 2). A portable chair was available for the participants to rest, if required, while performing the test and applying the pre- and post-test measures. The participants wore safety harnesses and were not allowed to grasp handrails during the test. A stopwatch was used for the 6MWT. No minimal or maximal speed limit was set for GRAIL; however, participants were instructed to walk naturally and not run. All participants completed the test without stopping.

### Outcome measures

The primary outcome measure was the distance walked (6MW_D_), which was assessed at the end of the 6MWT.^2^ The 6MW speed (6MW_S_) was also measured at the end of the test. According to the ATS,^2^ the following measurements were performed for the assessment of secondary outcomes at baseline after 10 min of rest and at the end of the 6MWT: SBP, DBP, HR (BTL Cardiopoint BTL-08; ABPM, UK), arterial oxygen saturation (SaO_2_; BPM-200; Biosys, Co., Ltd., Gangwon-do, South Korea), dyspnea, and overall fatigue. The participants were asked to rate their dyspnea and fatigue using the modified Borg scale, which consists of 11 points (ranging from 0 [nothing at all] to 10 [maximal]).

### Statistical analyses

Statistical analyses were performed using IBM SPSS Statistics version 24 for Windows (IBM Corporation, Armonk, NY, USA). Means and standard deviations were calculated for all continuous variables. Shapiro–Wilk's test and descriptive analysis were used to evaluate the data distribution. All continuous variables were normally distributed. A univariate general linear model analysis was performed, considering the control and intervention groups as fixed factors, in which the effect of the order of intervention was adjusted. This was performed for the following purposes: (a) to compare selected functional parameters obtained from the corridor-based 6MWT results with those from the GRAIL-based 6MWT results; and (b) to determine the possibility of applying the GRAIL-based 6MWT instead of the corridor-based 6MWT. Interventional comparisons were performed using the model and mean differences. Minimal detectable change at 90% confidence interval (CI; MDC_90_) was calculated using the following equation: $Standard\;error\;of\;measurement\times 1.69\times \sqrt{2}$. The correlation between the 6MW_D_ and 6MW_S_ was determined using Pearson's correlation coefficients. All data were considered statistically significant at *p* < 0.05.

## Results

### Demographic data

Of the 43 screened participants, 30 were enrolled and completed the study. Thirteen participants were excluded because they either did not meet the inclusion criteria or declined to participate (Figure 1). The mean (standard deviation) age, BMI, and respiratory rate of the participants were 20.6 (1.6) years, 22.1 (1.8) kg/m^2^, and 17.6 (3.2), respectively.

### Outcome measures

Table 1 shows the results of the primary and secondary outcome measures for both the GRAIL- and corridor-based 6MWTs. Both 6MW_D_ (mean difference [MD] = 56.5 m; 95% CI = 25.003, 87.957; *p* = 0.001) and 6MW_S_ (MD = 0.16 m/s; 95% CI = 0.065, 0.239; *p* = 0.001) were significantly higher for the corridor-based 6MWT than the GRAIL-based 6MWT. These differences reached the MDC_90_ for both the 6MW_D_ (37.5 m) and 6MW_S_ (0.10 m/s). No statistical differences (*p* ≥ 0.272) were observed between the corridor- and GRAIL-based 6MWTs in any of the secondary outcomes: SBP (MD [95% CI] = −0.93 [–5.416, 3.55]); DBP (2.23 [–2.25, 6.717]); SaO_2_ (0.63 [–0.51, 1.777]); HR (0.6 [–4.8, 6]); dyspnea (0.02 [–0.498, 0.464]); overall fatigue (−0.1 [–0.526, 0.326]). A strong positive correlation was observed between the 6MW_D_ and 6MW_S_ (*r* = 0.999; *p* < 0.001), indicating that the faster the walking speed, the longer the walking distance, and vice versa.

**Table 1:** Results of the GRAIL-based and corridor-based 6-minute walk test.

Variable	Test	Mean (SD)	Between-group mean difference (95% confidence interval)	*P*-value	SEM	MDC_90_
*Primary outcomes*						
6MW_D_ (m)	6MWT-C 6MWT-G	470.4 (58.8) 413.9 (61.9)	56.5∗ (25.003, 87.957)	0.001	15.719	37.5 m
6MW_S_ (m/s)	6MWT-C 6MWT-G	1.3 (0.2) 1.2 (0.2)	0.16∗ (0.065, 0.239)	0.001	0.043	0.10 m/s
*Secondary outcomes*						
SBP (mmHg) [pre/post]	6MWT-C 6MWT-G	125.8 (10.8)/125.1 (9.3) 123.4 (10.8)/126.0 (7.9)	−0.93 (−5.416, 3.550)	0.678	2.239	5.30 mmHg
DBP (mmHg) [pre/post]	6MWT-C 6MWT-G	75.5 (6.0)/76.6 (8.8) 73.0 (9.1)/74.3 (8.7)	2.23 (−2.250, 6.717)	0.323	2.239	5.30 mmHg
SaO_2_ (%) [pre/post]	6MWT-C 6MWT-G	98.8 (0.4)/98.9 (0.4) 98.8 (0.7)/98.2 (3.1)	0.63 (−0.510, 1.777)	0.272	0.571	1.351%
HR (b/min) [pre/post]	6MWT-C 6MWT-G	74.4 (10.2)/76.8 (11.31) 77.3 (8.5)/76.2 (9.38)	0.60 (−4.800, 6.000)	0.825	2.696	6.378 b/min
Dyspnea (0–10) [pre/post]	6MWT-C 6MWT-G	0.2 (0.5)/0.6 (1.0) 0.1 (0.4)/0.6 (0.9)	0.02 (−0.498, 0.464)	0.945	0.240	0.567
Overall fatigue (0–10) [pre/post]	6MWT-C 6MWT-G	0.1 (0.5)/0.5 (1.0) 0.1 (0.6)/0.6 (0.8)	−0.10 (−0.526, 0.326)	0.640	0.213	0.504

∗Statistically significant *P* ≤ 0.05 and clinically meaningful.

**Abbreviations:** 6MW_D_: 6-minute walk distance, 6MW_S_: 6-minute walk speed, 6MWT-C: 6-minute walk test using the corridor, 6MWT-G: 6-minute walk test using GRAIL, DBP: diastolic blood pressure, HR: heart rate, MDC_90:_ minimal detectable change at 90% confidence interval, SaO_2_: arterial oxygen saturation, SBP: systolic blood pressure, SD: standard deviation, SEM: standard error of measurement.

## Discussion

In this study, we compared the effects of use of GRAIL and a corridor to perform the 6MWT on the functional performance of healthy young adults. The corridor-based 6MWT resulted in higher distance (6MW_D_) and speed (6MW_S_) than the GRAIL-based 6MWT. No differences were observed in any of the other functional outcomes (SBP, DBP, SaO_2_, HR, dyspnea, or overall fatigue) between the two testing methods.

Our results showed that healthy young adults walked further in the corridor (470.4 m) than in the GRAIL (413.9 m). This finding is consistent with that of a previous study that included healthy elderly participants, where the effects of corridor walking were compared with those of walking on a non-motorized treadmill.^9^ However, the results of the present study are not consistent with the findings of Liu et al.,^5^ who reported that healthy elderly individuals walked further on the GRAIL (692 m) than on the corridor (668 m). In patients, the results regarding walking distance were contradictory. Liu et al.^5^ found that the 6MW_D_ was shorter on GRAIL (483 m) than on a corridor (511 m) for patients with COPD. However, the walking distance was shorter for corridor walking compared to walking on a regular treadmill for patients with COPD^10^ and cardiac disorders.^11^ In healthy middle-aged to old adults, no significant differences were observed in the distances walked in a level-ground 6MWT and on a self-paced regular treadmill.^8^ The discrepancies between our findings and those of other studies may be attributed to the differences in the characteristics of participants and the type of treadmill used.

In this study, young healthy participants walked faster in the corridor (1.3 m/s) than on GRAIL (1.2 m/s), with a mean difference of 0.16 m/s that exceeded the MDC (0.1 m/s). In contrast, Liu et al.^5^ found no difference in 6MW_S_ between the GRAIL- and corridor-based 6MWTs (both 1.9 m/s) for healthy elderly individuals. One potential explanation for the discrepancies between our findings and those of Liu et al. could be differences in participant characteristics such as age. In patients with COPD, the 6MW_S_ was higher in the corridor (1.4 m/s) than in the GRAIL (1.3 m/s).^5^ However, the authors did not discuss the findings of speed-related analysis in their report. In situations other than the 6MWT, few studies have compared the gait speeds between corridor walking and GRAIL-based walking for healthy young participants. Mohler et al.^12^ reported lower preferred walking speeds with GRAIL than with corridor walking. By contrast, Plotnik et al.^13^ found that gait speed with GRAIL was comparable to that in the corridor condition, although reaching a constant gait velocity required a longer duration on a self-paced treadmill than that for corridor walking. One of the advantages of self-paced treadmill walking over fixed-speed treadmill walking is that self-paced treadmill walking offers a natural way of controlling and varying the walking speed, possibly leading to a more natural gait than that achieved with fixed-speed treadmill walking.^3^

In the present study, a strong positive correlation was found between the 6MW_S_ and 6MW_D_. This could explain the proportional increase in both outcomes. The differences between the corridor- and GRAIL-based 6MWTs in terms of the 6MW_D_ and 6MW_S_ may be attributed to different factors. For example, GRAIL-based walking may require more effort than ground walking, resulting in different levels of energy expenditure for each participant. In addition, adaptability toward learning new tasks or unfamiliarity with the task of walking on GRAIL might have affected the participants.^5^ Moreover, the participants were more familiar with walking in a corridor than on GRAIL, although they had practiced walking on GRAIL before the actual test.^11^ Furthermore, the participants in the current study wore safety harnesses during GRAIL. This mechanical pulling of the harness may have caused the participants to exert greater effort in walking on GRAIL than that exerted during corridor walking, which consequently made them walk less on GRAIL than in the corridor.^11^

None of the secondary outcome measures in the current study (SBP, DBP, SaO_2_, HR, dyspnea, or overall fatigue) differed between the corridor- and GRAIL-based 6MWTs. These results are supported by those of Liu et al.^5^ who demonstrated no differences in the degree of dyspnea or fatigue between the two methods in healthy elderly individuals. By contrast, Janaudis-Ferreira et al.^9^ found greater levels of dyspnea and fatigue during walking on a non-motorized treadmill than during the corridor-based 6MWT in healthy elderly participants. Liu et al.^5^ found higher HRs after the corridor-based 6MWT than after the GRAIL-based 6MWT. Studies on patients with cardiac and pulmonary disorders have demonstrated contradictory results, such as no differences in dyspnea or HR between corridor and treadmill tests^10^ and less dyspnea and fatigue on the GRAIL.^5^ These discrepancies may be due to factors such as differences in participant characteristics (e.g., age and health status), methodologies employed (e.g., specific exercise protocols and outcome measures used), and equipment utilized.

### Clinical implications

The results of the present study show that the results of corridor- and GRAIL-based 6MWTs are not interchangeable for healthy young adults, which is supported by previous studies conducted in healthy elderly individuals.^5^^,^^9^ Furthermore, these findings suggest that walking distance and speed during either of the tests cannot be predicted. Clinicians and researchers may use the GRAIL system as an alternative to the corridor, particularly when a 30-m corridor is not available. Although this study was performed in healthy individuals, the findings may have implications for the patient population. In addition, the implementation of GRAIL-based testing in clinical practice presents challenges including the potential upfront cost of equipment, such as eye tracking systems or virtual reality setups, unlike for traditional methods. Additionally, healthcare professionals may require specialized training to effectively conduct and interpret the results of GRAIL-based tests. Finally, ensuring access to technical support for maintenance of equipment and troubleshooting is crucial for smooth implementation of GRAIL-based tests.

### Strengths and limitations

The cross-over design of the present study is the main strength of this study. In this design, the effects of the treatments were compared for each participant, each of whom served as the control, consequently eliminating interparticipant variability and reducing the effects of covariates. Second, the cross-over design allowed high power and statistical efficiency because we could obtain estimates with similar levels of accuracy, even though the sample size was small.^14^ Although the randomized cross-over design is a good choice for comparing the effects of the two methods, carryover effects are a concern. Even with a washout period, the effects of one test may persist and influence those of the other test. This is particularly true because no study has determined the ideal washout durations for similar interventions. However, determining the perfect washout period can be difficult, and its effectiveness can vary depending on the intervention.^14^ This study had a couple of limitations. First, the participants were men of a restricted age category. Therefore, our findings should not be generalized to populations with other characteristics. Second, the balance may have been affected during the GRAIL-based 6MWT. However, GRAIL can be used to continuously assess balance and other gait qualities during virtual reality and self-paced walking.

## Conclusions

Although no differences were observed in terms of secondary outcome measures between corridor- and GRAIL-based MWTs, young healthy participants walked further and faster in the corridor-based 6MWT than in the GRAIL-based 6MWT. This indicates that neither of the testing methods can be used interchangeably. However, GRAIL may be used as an alternative to corridor testing, particularly if a 30-m space is not available. Further research is required to gain a comprehensive understanding of the potential benefits of the GRAIL-based 6MWT. This involves addressing the limitations of the current study and incorporating two key elements: (a) long-term follow-up assessments to offer valuable insights into the sustainability of any differences in physiological responses; and (b) gait parameter analysis to provide detailed information about walking mechanics.

## Source of funding

The authors received no financial support for the research, authorship, or publication of this article.

## Conflict of interest

The authors have no conflict of interest to declare.

## Ethical approval

This study was approved by the institutional review board of Imam Abdulrahman Bin Faisal University (IRB-UGS-2015-03-235; date: 27/12/2015).

## Consent

Written informed consent was obtained from all participants.

## Authors contributions

MAA and QIM conceived and designed the study. MAA, SSA, HMA, MAA, AAA, HAS, ASA, and MA collected data. MAA and AMA analyzed and interpreted data and wrote the initial and final drafts of the article. All authors have critically reviewed and approved the final draft and are responsible for the content and similarity index of the manuscript.
